# Arthroscopic fixation techniques for tibial eminence fractures in pediatric patients: a review

**DOI:** 10.3389/fped.2024.1347637

**Published:** 2024-03-26

**Authors:** Canfeng Li, Xiancheng Huang, Qingjun Yang, Yong Luo, Jiatong Li, Sufen Ye, Wenqian Lu, Xintao Zhang, Tian You

**Affiliations:** ^1^ Sports Medicine and Rehabilitation Center, Peking University Shenzhen Hospital, Shenzhen, China; ^2^ Clinical Medical College, Weifang Medical University, Weifang, China; ^3^ Clinical Medical College, Shantou University Medical College, Shantou, China; ^4^ Clinical Medical College, Shenzhen University, Shenzhen, China

**Keywords:** arthroscopic fixation, tibial eminence, tibial spine, avulsion, suture bridge

## Abstract

The introduction of new internal fixation devices and arthroscopic techniques has led to significant changes in the surgical treatment of tibial eminence fractures (TEFs) in children. In recent years, arthroscopic surgery has arisen as the gold standard for the treatment of TEFs. This popularity of arthroscopic techniques has reduced surgical complications and improved patient prognosis. In this paper, we investigate the current situation of the use of arthroscopic fixation techniques for pediatric TEFs. We searched the PubMed database using the terms “arthroscopic treatment and tibial eminence,” “arthroscopic treatment and tibial spine,” “tibial eminence avulsion”, “tibial spine fracture”, with no limit on the year of publication. From these articles, we reviewed the use of various arthroscopic TEFs fixation techniques reported in the current literature. Overall, we found that the choice of fixation method seems to have no effect on clinical outcomes or imaging results. However, if an easy, strong fixation that is less prone to epiphyseal damage is desired, as a junior practitioner, the anchor technique should be mastered first, whereas for senior practitioners, a variety of fixation techniques for TEFs should be mastered, including anchors, sutures, and screws, so that personalized fixation can be achieved with the least amount of trauma, operative time, and complications. Higher quality studies are needed in the future to provide Useful evidence to determine the optimal fixation technique in terms of clinical outcomes, function, and complications.

## 1 Introduction

**Figure 1:**
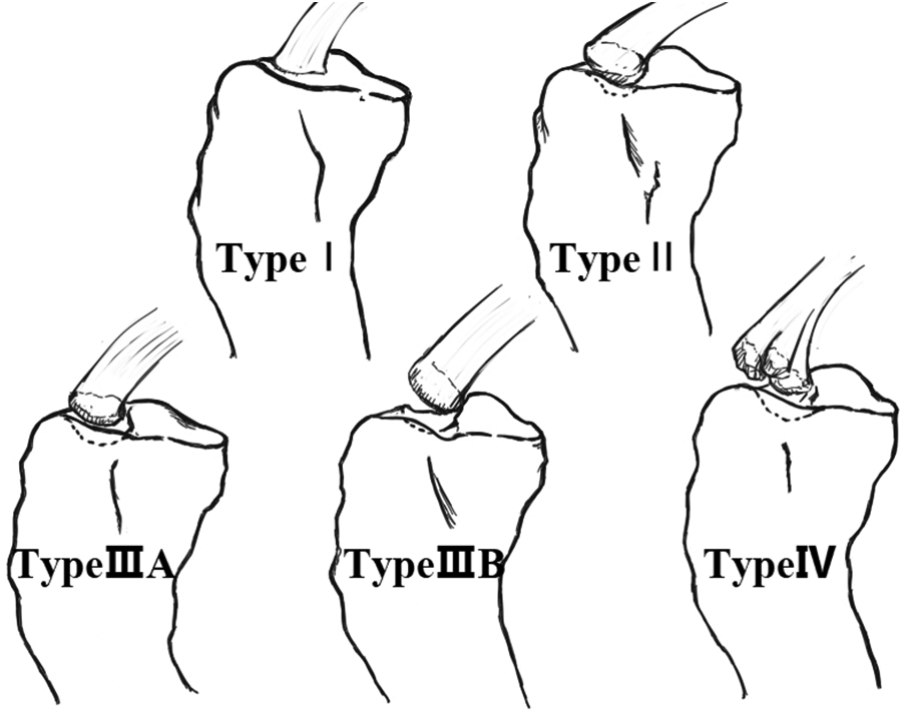
The classification of TEFs (Dr. Wei Dai redrew the picture.).

Tibial eminence fractures (TEFs), a serious intra-articular injury of the knee joint in children equivalent to the rupture of anterior cruciate ligament (ACL) in adults, was first described by Poncet in 1875 (1). This injury commonly occurs during a fall from a bicycle or during a knee hyperextension with valgus external rotation maneuver during sports activities, and results in fractures of the intercondylar spine of the tibia that are not fully ossified. These injuries are most prevalent in boys aged 8–14 years old (2–4). In rare cases, TEFs may also occur as a result of direct trauma or hyperextension of the knee (5). In 1959, Meyers and McKeever were the first to propose the typing of TEFs, with their classification still widely used today (6). Type I lesions are undisplaced or minimally displaced fractures involving the anterior margin of the spine; Type II fractures present with a superior displacement of the anterior part of the fragment, with the posterior portion still attached to the rest of the proximal tibia (termed the “bird's beak” pattern); in Type III lesions the fragment is completely detached. Type III fractures can be further divided into Type IIIA, in which only the ACL insertion is involved, and Type IIIB, in which the whole tibial eminence is involved (7). In addition, the Type IV (Figure 1) refers to the displacement and comminution of fracture fragments (8). Of these different types, type I fractures can be treated with closed reduction by hyperextension of the knee, whereas type II or type III fractures, for which closed reduction fails, require surgical treatment. Recently, Green et al. (9) introduced a new MRI-based classification, which evaluates fractures quantitatively based on the degree of fragment displacement and tissue involvement. This system was shown to help surgeons to make clinical decisions. Arthroscopic techniques are gradually becoming the gold standard for the surgical treatment of TEFs due to the higher post-operative pain, soft-tissue damage, and delay in rehabilitation of the open surgery. Besides, arthroscopy has several advantages, including that it allows for direct visualization of intra-articular injuries, the ability to accurately reduct the fracture fragments, and the simultaneous management of other concomitant intra-articular injuries (10).

We searched the PubMed database using the terms “arthroscopic treatment and tibial eminence,” “arthroscopic treatment and tibial spine,” “tibial eminence avulsion”, “tibial spine fracture”, with no limit on the year of publication. From these articles, we reviewed the use of various arthroscopic TEFs fixation techniques reported in the current literature.

Currently, there are no high-quality prospective randomized controlled trials to indicate the optimal fixation technique for TEFs. As such, in this study, we reviewed arthroscopic techniques and fixation methods for TEFs in children and adolescents with the aim of informing surgeons on the optimal treatment modality.

## 2 Kirschner wires

Arthroscopic Kirschner wire fixation of TEFs is relatively poorly documented, with only one article in German. In this article, the authors arthroscopically reduct the fracture fragments, followed by fixation with Kirschner wire or cannulated screw; all 19 minor patients regained normal range of motion without pain or epiphyseal injury. However, three patients experienced residual mild instability compared to the healthy side, but with no secondary cartilage or meniscus injury; one patient had a malunion of fracture (shifting up about 2 mm), but with no intercondylar notch impingement or impaired knee extension (11). The paper did not give the number of cases or results of fixation with Kirschner wires alone. Although the smooth surface and smaller diameter of Kirschner wire made it possible to avoid epiphyseal injury, the disadvantages of backing out of the pins, instable fixation, intercondylar notch impingement, and interference to MRI have prevented later authors from choosing this method.

## 3 Cannulated screws

Cannulated screws are the classic method of fracture fixation. Their use in the arthroscopic fixation of TEFs was first reported in 2002, by Reynders et al. (12); instead of passing through the center of the fracture fragments, the cannulated screws were drilled through the anterior edge of the fractures, and the screw tails and the washer were utilized to hold down the fragments. This resulted in instable fixation, loosening of the fragments (81.25%, 13/16), and susceptibility to knee extension deficits (25%, 4/16). With the advancement of arthroscopic techniques, an increasing number of surgeons have begun to use cannulated screws to penetrate the fracture fragments for arthroscopic fixation, and although there is still some laxity in the joint or intercondylar notch impingement post-operatively, most of the patients still regained a reasonable level of knee function (13–17). In addition, in 2018, Shin et al. (18) found that in their 27 patients who underwent screw fixation, the affected limb was on average 6.2 mm longer than the healthy side postoperatively. In conclusion, the biomechanics of cannulated screw fixation of TEFs are excellent, however, the screw may cause fragmentation of the fractures, popliteal neurovascular injury, and intercondylar notch impingement, ultimately resulting in the need for a second surgery to remove the internal fixation in some patients.

## 4 Pull-out sutures

Sutures with bone tunnel fixation was introduced as a method to overcome the complications of other techniques, including the intercondylar notch impingement or the interference to MRI. In 2005, Ahn et al. (19) reported a cohort of 14 cases of TEFs, all of which were fixed with sutures through the bone tunnels. All patients achieved complete healing of the fracture and a normal range of motion, and one case showed mild laxity (2 mm anterior shift on the KT-2000 test). In this study, two in five adolescents were found to have an affected limb that was 10 mm longer than the healthy side at final follow-up. Because pull-out sutures are easier, many other authors have taken this approach and obtained good results, with some authors knotting the sutures pulled out of the tibial tunnel at the tunnel opening outside the joint (20, 21), or fixing them outside the joint with partially threaded cancellous bone screws (6.5 mm) (22), or by knotting the suture after passing it through a 3-hole titanium plate (23). In general, the more sutures with tibial tunnels, the better the fracture reduction, particularly in those fragmented fractures. However, in children and adolescents with immature epiphysis, more tunnels are more prone to epiphyseal injury; hence, two to three small tunnels are more appropriate.

## 5 Metallic suture

Osti et al. (24) first reported an arthroscopic technique in which the tibial tunnel was passed with a metallic suture and then fixed to the distal tibia with a cortical bone screw. All 10 patients in their cohort achieved fracture healing at six to eight weeks postoperatively and returned to sports at six to nine months postoperatively. Overall, the prognosis was excellent/good and fair/poor in 80% and 20% of patients, respectively. However, the patients in this study were 17–41 years old, and therefore, it is speculated that these are all cases of mature epiphysis. Thus, whether this technique can be used in children and adolescents with unclosed epiphyses remains unclear.

## 6 Meniscus arrows

Meniscus Arrows is an absorbable material, which can be considered as the smallest absorbable screw in diameter (1.1 mm); it has a resistance to pullout force of 68 N for single one and a pullout force of 122 N for three pieces (25). This material was used for the fixation of TEFs in one study, which found that the fractures of all 12 children healed successfully without displacement or limitation of joint motion, allowing return to sports (26).

## 7 Anchors

The anchor technique has been extensively used and continuously improved in shoulder injuries such as glenoid labral tears and rotator cuff tears, and eventually becoming the standard fixation technique for shoulder arthroscopy with increasing application in other areas of orthopedics. The different types of anchor technique include single-row fixation and the double-row suture bridge. In 2008, In et al. (27) were the first to use four absorbable anchors for arthroscopic fixation of TEFs in patients with comminuted fractures and immature epiphysis with favorable results. In the same year, Vega et al. (28) adopted a similar technique for fixation of TEFs (6 adolescents, 1 adult), with the important difference that Vega et al. used only one metal anchor. In this study, the anterior drawer test, Lachaman test, and pivot shift were negative, the mean distance of anterior tibial shift was 2 mm (range 1–3 mm), and all the patients returned to the pre-injury level of sport at the final follow-up. In 2012, Mann et al. (29) pioneered the introduction of the double-row suture bridge to the treatment of TEFs, which was first used for larger rotator cuff tears and later for the treatment of avulsion fractures of the greater tuberosity of the humerus. The authors concluded that this technique is suitable for all types of TEFs, and is able to apply uniform planar compression to the avulsed fracture fragments rather than the point compression fixation from a single row of anchor, thereby promoting fracture healing and preventing fragmentation. Although Mann et al. did not state whether their case was adolescents or not, this technique was quickly recognized and widely accepted, with several variations of the procedure ultimately being derived (30–34). Overall, due to the increasing popularity of arthroscopic manipulation techniques, the fact that anchor technique results in little damage to the epiphysis and fixation is very stable, it has gained popularity in recent years, showing the potential to become a mainstream surgery in the future.

## 8 Comparison of different fixation techniques

### 8.1 Biomechanical comparison

Several prior studies in animal models have been conducted to compare the different arthroscopic techniques used to treat TEFs. In one study, Sawyer et al. (35) randomly divided the fractured knees of 24 young pigs into three treatment groups: the suture group, the cannulated screw group, and the PushLock anchor group. After the manipulation, all specimens were subjected to two stages of biomechanical testing: cyclic dynamic testing and the ultimate tensile load test. The results showed no statistically significant difference between the results of the cyclic dynamic testing between the three groups (*P* = 0.412). For the ultimate load test, there was no difference between the screw (183 ± 82.5 N, *P* = 0.007) and suture groups (199 ± 55.8 N, *P* = 0.017); however, the PushLock group (340 ± 117 N) was subjected to significantly higher mean ultimate failure loads. Therefore, the authors concluded that this suture bridge provided better results in terms of ultimate failure loads compared with the cannulated screw group and suture fixation, and that this technique therefore deserves clinical promotion.

In another study, Hapa et al. (36) divided 49 adult sheep knees equally into seven groups: group 1 (No. 2 FiberWire suture), group 2 (No. 2 UltraBraid suture), group 3 (No. 2 MaxBraid suture), group 4 (No. 2 Hi-Fi suture), group 5 (No. 2 OrthoCord suture), group 6 (titanium anchor), and Group 7 (EndoButton + No. 2 FiberWire); the specimens were then subjected to cyclic dynamic testing and the ultimate tensile load test. The results of this analysis revealed that after the initial 100 cycles of testing, Group 7 had the smallest displacement of (1.8 ± 1.2) mm, which was statistically significant. However, when a comparison was made after 100 cycles, there was no statistical difference among the seven groups. In this study, the authors defined failure of the ultimate tensile load test as three conditions: suture rupture, suture cutting into the bone, and ACL rupture, of which the most frequent in the seven groups was suture rupture, with the highest ultimate tensile load in group 7. Therefore, the authors concluded that fixation of TEFs with the EndoButton can achieve greater initial fixation strength than suture anchors or various high-strength sutures. However, it is worth noting that the only one of ACL rupture occurred in the EndoButton group, which would make revision surgery more difficult in real world scenarios. This is because, in the case of suture rupture or suture cutting through the bone, revision surgery with other internal fixation techniques are strong enough for chronic TEFs; whereas, in the case of ligament rupture, it must undergo anterior cruciate ligament reconstruction with greater damage and slower rehabilitation.

In a cadaveric study, Li et al. (37) divided the knee joints of 24 adult cadavers equally into four groups: group A (screws), group B (traditional FiberWire suture), group C (FiberWire neckwear knots technique), and group D (suture bridge); the specimens were then subjected to cyclic dynamic testing (100 N, 500 cycles) and the single tensile failure test. All four groups passed the cyclic dynamic test, with group C having the highest ultimate tensile load (*P* < 0.05) and group D having the smallest displacement distance (*P* < 0.05). In conclusion, the authors noted that both the neckwear knots technique and the suture bridge technique showed superior biomechanical properties, with the former having the highest ultimate tensile load and the latter having the smallest displacement after undergoing cyclic loading, indicating it could be a good choice for the treatment of TEFs.

However, the above study specimens were not representative of the characteristics of children's skeleton, Johnstone et al. (38) matched the age and side of 12 knees from 6 children's cadavers, and divided them into a double screw group and the suture group, in which the double screw group was fixed with two 4 mm × 35 mm partially threaded cannulated screws, and the suture group was fixed with two No. 2 FiberWire sutures through the anterior 1/3 and posterior 1/3 of the cruciate ligament, and then pulled through the bone tunnels on the medial and lateral side of the fracture fragment to be knotted and fixed outside the joint. Overall, this study found no major differences in ultimate loads between the two groups; although the double screw group showed increased ligament stiffness and decreased stretch compared to those in the suture group, these differences did not reach significance. The authors concluded that the screw and suture techniques have similar biomechanical properties in the treatment of TEFs. In addition, the pediatric TEFs model had lower ultimate loads, and was more prone to failure than the adult cadaveric and porcine bone specimens used in previous studies. Unfortunately, due to the limited number of pediatric cadaveric specimens, the authors did not test the single-row fixation or double-row suture bridge technique, which have been widely recognized as useful in greater tuberosity fractures of the shoulder.

### 8.2 Comparison of clinical studies

Callanan et al. (39) reviewed the outcomes of 68 children with TEFs, classified as Meyer & McKeever type II or III, followed up for at least 12 months. There were 33 cases in the suture group and 35 cases in the screw group, with no inter-group differences in the seven items of postsurgical arthrofibrosis, ACL reconstruction, meniscal procedures, instability, range of motion, return to sport, or time to return to sport. Although the fracture fragments were found to be elevated a significantly greater distance on postoperative imaging in the suture group (5.4 vs. 3.5 mm; *P* = 0.005), this did not affect prognosis. In addition, the screw group had a higher incidence of reoperations (13 vs. 23; *P* = 0.03), with a reoperation rate nearly three times higher than that of the suture group (OR: 2.9; *P* = 0.03). The authors conclude that the clinical outcomes of both suture and screw fixation techniques are basically the same, and that postoperative elevation of the fracture fragments does not affect the surgical outcome. Overall, the authors supported the use of suture fixation, especially in the case of comminuted fracture fragments, given the greater likelihood of a second surgery (planned or unplanned) after screw fixation and the avoidance of postoperative MRI artifacts.

In 2022, Jain et al. (40) published a prospective adult-based randomized controlled trial in which the authors concluded that suture transosseous tunnel fixation was superior to screw fixation in the treatment of TEFs, with better clinical and functional outcomes and a lower chance of secondary surgery.

### 8.3 Systematic evaluations

Several systematic evaluations investigating the optimal arthroscopic technique for TEFs have also been published. In 2016, Osti et al. (41) published a systematic evaluation in which the authors included 24 articles, not strictly limited to adolescents, after an extensive search of the literature in Italian and English, including 13 retrospective and 11 prospective studies. The authors concluded that the arthroscopic technique was superior to the open technique, achieving reductions in the incidence of postoperative pain and infection, in addition to shortening the length of hospital stay. Analysis of the clinical and imaging findings revealed no difference between the various fixation methods available at the time, and the authors recommended that surgeons choose the appropriate fixation technique based on their own experience.

In 2022, Chang et al. (42) performed a meta-analysis of studies investigating screw vs. suture fixation; after an extensive search of the English language literature, the authors included only five retrospective cohort studies (level 3 evidence), not strictly limited to adolescents. The results showed that there was no significant difference in clinical outcome scores between screw and suture fixation, but there was a higher risk of requiring a second operation due to complications after screw fixation (RR: 2.33) and the need to remove the internal fixation (RR 8.52), especially when used in children with a non-closed epiphysis.

Unlike previous researchers, in 2023, Limone et al. (5) systematically evaluated 12 articles exclusively on TEFs in children and adolescents (age <16 years) after an extensive search of the English language literature. They included predominantly retrospective studies comprising a total of 381 knees, in patients (222 males and 142 females) with a mean age of 12.1 years, and a mean follow-up of 45.7 months. The article focused on comparing screw fixation with suture fixation; however, due to heterogeneity between studies, articles on the two types of fixation were not comparable in terms of follow-up time, age, trauma etiology, and time of injury (*P* < 0.01). The results showed that the suture fixation technique was superior to screw fixation in terms of Tegner, IKDC, and Lysholm scores (*P* < 0.001), and that the need for a second surgery to remove the internal fixation was lower (*P* < 0.001), but there was a higher chance of developing arthrofibrosis postoperatively (*P* < 0.05).

Unfortunately, the above literature did not provide a clear answer regarding which fixation technique has the best efficacy, but we can consider it in the context of knee fibrosis, which is the most common postoperative complication (5). The Tibial Spine Research Interest Group showed that the risk factors for arthrofibrosis include prolonged braking time after surgery, combined ACL injury, age younger than 10 years, more severe injury, delayed time from injury to surgery, and screw fixation (compared to suture fixation) (43). Therefore, the optimal approach should be easy, strong, and less prone to epiphyseal damage technique, which would reduce operative time and immobilization time, or even eliminate the need for immobilization postoperatively. Considering this aspect, together with the previous biomechanical analysis, it is reasonable to assume that the double-row suture bridge is a sufficiently secure and easy to perform technique that is essentially free of epiphyseal damage and deserves to be emphasized, as has been demonstrated in arthroscopic surgery of the shoulder joint (44).

## 9 Conclusion

The development of arthroscopic techniques and new types of fixation has led to a significant number of innovations in the surgical treatment of TEFs in children and adolescents, and due to the paucity of high-quality, prospective, randomized controlled studies, it is not possible to specify which fixation technique has the best efficacy. The advantages and disadvantages of these six techniques were in Table 1. Overall, anchor fixation can be applied to all types of fractures and is not prone to metaphyseal injury; for type II fractures, a single row of anchors is enough, whereas for type III fractures with significant displacement, the presence of entrapment, or where the posterior hinge has broken, the double-row of anchors should be used in the suture bridge technique. Conversely, in cases where the fracture fragment is large and thick and the epiphysis is almost mature, cannulated screws may be a easier option. Therefore, we recommend that junior doctors should initially focus on mastering the anchor technique, while senior doctors should gain experience with a variety of TEFs fixation techniques, including anchors, sutures, and screws, in order to achieve personalized fixation with minimal trauma, shortest operative time, and the lowest rate of complications.

**Table 1:** The advantages and disadvantages of these six techniques.

	Advantages	Disadvantages
Kirschner wires	Avoid epiphyseal injury.	Backing out of the pins. Instable fixation. Intercondylar notch impingement. Interference to MRI.
Cannulated screws	Easy to learn. Good biomechanics performance.	Causing fragmentation of the fractures. Popliteal neurovascular injury. Intercondylar notch impingement. Interference to MRI. A higher incidence of reoperations.
Pull-out sutures	Overcoming the intercondylar notch impingement or the interference to MRI. Good biomechanics performance.	More tibial tunnels are more prone to epiphyseal injury. A higher chance of developing arthrofibrosis postoperatively.
Metallic suture	It is not yet clear whether this technology can be used for children and adolescents with unclosed epiphyses.	
Meniscus Arrows	Absorbable internal fixation with the smallest diameter. Good biomechanics performance.	Causing fragmentation of the fractures. Intercondylar notch impingement.
Anchors	Little damage to the epiphysis. Stable fixation and good biomechanics performance. A very mature arthroscopic technique. The planar compression to the fractures which can promote the healing and prevent fragmentation.	There is a learning curve. Relatively expensive.

## References

[1] Kendall NS Hsu SY Chan KM. Fracture of the tibial spine in adults and children. A review of 31 cases. J Bone Joint Surg Br. (1992) 74:848–52. 10.1302/0301-620X.74B6.1447245 1447245

[2] Wiktor Ł Tomaszewski R. Results of anterior cruciate ligament avulsion fracture by treatment using bioabsorbable nails in children and adolescents. Children (Basel). (2022) 9:1897. 10.3390/children9121897 36553339 PMC9776932

[3] DeFrancesco CJ Wilson L Lebrun DG Memtsoudis SG Fabricant PD. Pediatric tibial spine fractures: exploring case burden by age and sex. Orthop J Sports Med. (2021) 9:23259671211027237. 10.1177/23259671211027237 34552990 PMC8450686

[4] Axibal DP Mitchell JJ Mayo MH Chahla J Dean CS Palmer CE Epidemiology of anterior tibial spine fractures in young patients: a retrospective cohort study of 122 cases. J Pediatr Orthop. (2019) 39:e87–90. 10.1097/BPO.0000000000001080 28945690

[5] Limone B Zambianchi F Cacciola G Seracchioli S Catani F Tarallo L. Management and outcomes of tibial eminence fractures in the pediatric population: a systematic review. Children (Basel). (2023) 10:1379. 10.3390/children10081379 37628378 PMC10453829

[6] Meyers MH Mckeever FM. Fracture of the intercondylar eminence of the tibia. J Bone Joint Surg, (Am.). (1959) 41-a:209–20; discussion 220–2. 10.2106/00004623-195941020-00002 13630956

[7] Jackson TJ Storey EP Ganley TJ, Tibial Spine Interest Group. The surgical management of tibial spine fractures in children: a survey of the Pediatric Orthopaedic Society of North America (POSNA). J Pediatr Orthop. (2019) 39:e572–7. 10.1097/BPO.0000000000001073 31393291

[8] Zaricznyj B. Avulsion fracture of the tibial eminence: treatment by open reduction and pinning. J Bone Joint Surg Am. (1977) 59:1111–4. 10.2106/00004623-197759080-00022 591548

[9] Green D Tuca M Luderowski E Gausden E Goodbody C Konin G. A new, MRI-based classification system for tibial spine fractures changes clinical treatment recommendations when compared to myers and mckeever. Knee Surg Sports Traumatol Arthrosc. (2019) 27:86–92. 10.1007/s00167-018-5039-7 29961096

[10] Kelly S DeFroda S Nuelle CW. Arthroscopic assisted anterior cruciate ligament tibial spine avulsion reduction and cortical button fixation. Arthrosc Tech. (2023) 12:e1033–8. 10.1016/j.eats.2023.02.052 37533906 PMC10390881

[11] Sommerfeldt DW. Die arthroskopisch assistierte refixation bei knöchernen ausrissen des vorderen kreuzbands im kindes- und jugendalter [Arthroscopically assisted internal fixation of avulsion fractures of the anterior cruciate ligament during childhood and adolescence]. Oper Orthop Traumatol. German. (2008) 20:310–20. 10.1007/s00064-008-1403-y 19169775

[12] Reynders P Reynders K Broos P. Pediatric and adolescent tibial eminence fractures: arthroscopic cannulated screw fixation. J Trauma. (2002) 53:49–54. 10.1097/00005373-200207000-00011 12131389

[13] Kocher MS Foreman ES Micheli LJ. Laxity and functional outcome after arthroscopic reduction and internal fixation of displaced tibial spine fractures in children. Arthroscopy. (2003) 19:1085–90. 10.1016/j.arthro.2003.10.014 14673450

[14] Pan RY Yang JJ Chang JH Shen HC Lin LC Lian YT. Clinical outcome of arthroscopic fixation of anterior tibial eminence avulsion fractures in skeletally mature patients: a comparison of suture and screw fixation technique. J Trauma Acute Care Surg. (2012) 72:E88–93. 10.1097/TA.0b013e3182319d5a 22328000

[15] Bomar JD Edmonds EW. Surgical reduction and fixation of tibial spine fractures in children: arthroscopic suture fixation. JBJS Essent Surg Tech. (2016) 6:e17. 10.2106/JBJS.ST.15.00053 30237926 PMC6145633

[16] Hiranaka T Furumatsu T Tanaka T Okazaki Y Kodama Y Kamatsuki Y Combining pullout suture and retrograde screw fixation for anterior cruciate ligament tibial eminence avulsion fractures: a case report. J Orthop Surg (Hong Kong. (2020) 28:2309499020918681. 10.1177/2309499020918681 32489139

[17] Zheng C Han H Cao Y. Arthroscopically assisted cannulated screw fixation for treating type III tibial intercondylar eminence fractures: a short-term retrospective controlled study. Front Surg. (2021) 8:639270. 10.3389/fsurg.2021.639270 34239891 PMC8259787

[18] Shin CH Lee DJ Choi IH Cho TJ Yoo WJ. Clinical and radiological outcomes of arthroscopically assisted cannulated screw fixation for tibial eminence fracture in children and adolescents. BMC Musculoskelet Disord. (2018) 19:41. 10.1186/s12891-018-1960-7 29409477 PMC5801812

[19] Ahn JH Yoo JC. Clinical outcome of arthroscopic reduction and suture for displaced acute and chronic tibial spine fractures. Knee Surg Sports Traumatol Arthrosc. (2005) 13:116–21. 10.1007/s00167-004-0540-6 15756616

[20] Ochiai S Hagino T Watanabe Y Senga S Haro H. One strategy for arthroscopic suture fixation of tibial intercondylar eminence fractures using the meniscal viper repair system. Sports Med Arthrosc Rehabil Ther Technol. (2011) 3:17. 10.1186/1758-2555-3-17 21831294 PMC3163208

[21] Zhao C Bi Q Bi M. Management of a type two avulsion fracture of the tibial intercondylar eminence in children: arthroscopic suture fixation versus conservative immobilization. Int Orthop. (2018) 42:1363–9. 10.1007/s00264-018-3855-5 29516235

[22] Mittal R Digge V Selvanayagam R. Subintermeniscal ligament pullout suture technique for anterior cruciate ligament avulsion fracture fixation-AIIMS technique. J Knee Surg. (2021) 34:1355–8. 10.1055/s-0040-1709180 32330973

[23] Maliwankul K Chuaychoosakoon C. Suturing the anterior cruciate ligament using a No. 16 intravenous catheter needle in avulsion anterior cruciate ligament injury. Arthrosc Tech. (2020) 9:e1191–6. 10.1016/j.eats.2020.04.019 32874900 PMC7451439

[24] Osti L Merlo F Liu SH Bocchi L. A simple modified arthroscopic procedure for fixation of displaced tibial eminence fractures. Arthroscopy. (2000) 16:379–82. 10.1016/s0749-8063(00)90082-3 10802475

[25] Wouters DB Bos RR Mouton LJ van Horn JR. The meniscus arrow or metal screw for treatment of osteochondritis dissecans? In vitro comparison of their effectiveness. Knee Surg Sports Traumatol Arthrosc. (2004) 12:52–7. 10.1007/s00167-003-0435-y 14586489

[26] Wouters DB de Graaf JS Hemmer PH Burgerhof JG Kramer WL. The arthroscopic treatment of displaced tibial spine fractures in children and adolescents using meniscus arrows®. Knee Surg Sports Traumatol Arthrosc. (2011) 19:736–9. 10.1007/s00167-010-1341-8 21153538 PMC3076577

[27] In Y Kim JM Woo YK Choi NY Moon CW Kim MW. Arthroscopic fixation of anterior cruciate ligament tibial avulsion fractures using bioabsorbable suture anchors. Knee Surg Sports Traumatol Arthrosc. (2008) 16:286–9. 10.1007/s00167-007-0466-x 18157488

[28] Vega JR Irribarra LA Baar AK Iñiguez M Salgado M Gana N. Arthroscopic fixation of displaced tibial eminence fractures: a new growth plate-sparing method. Arthroscopy. (2008) 24:1239–43. 10.1016/j.arthro.2008.07.007 18971053

[29] Mann MA Desy NM Martineau PA. A new procedure for tibial spine avulsion fracture fixation. Knee Surg Sports Traumatol Arthrosc. (2012) 20:2395–8. 10.1007/s00167-012-1906-9 22270677

[30] Sawyer GA Hulstyn MJ Anderson BC Schiller J. Arthroscopic suture bridge fixation of tibial intercondylar eminence fractures. Arthrosc Tech. (2013) 2:e315–8. 10.1016/j.eats.2013.04.004 24400173 PMC3882679

[31] Li J Liu C Li Z Fu Y Yang Y Zhang Q. Arthroscopic fixation for tibial eminence fractures: comparison of double-row and transosseous anchor knot fixation techniques with suture anchors. Med Sci Monit. (2018) 24:7348–56. 10.12659/MSM.912961 30318505 PMC6198712

[32] Fox JC Saper MG. Arthroscopic suture fixation of comminuted tibial eminence fractures: hybrid all-epiphyseal bone tunnel and knotless anchor technique. Arthrosc Tech. (2019) 8:e1283–8. 10.1016/j.eats.2019.06.018-e1288 31890496 PMC6926312

[33] Elqirem Z Alhanbali M Sbieh Y. Double-row fixation for avulsion of anterior cruciate ligament. Arthrosc Tech. (2019) 8:e1473–7. 10.1016/j.eats.2019.07.030 31890525 PMC6928369

[34] Yu D Yu R Zhang J Chen T Zhang B. Arthroscopic treatment of adult displaced tibial eminence fractures with anchor and pushlock fixation. Med (Baltim). (2020) 99:e21237. 10.1097/MD.0000000000021237 PMC7505351 32957304

[35] Sawyer GA Anderson BC Paller D Schiller J Eberson CP Hulstyn M. Biomechanical analysis of suture bridge fixation for tibial eminence fractures. Arthroscopy. (2012) 28:1533–9. 10.1016/j.arthro.2012.02.020 22607830

[36] Hapa O Barber FA Süner G Özden R Davul S Bozdağ E Biomechanical comparison of tibial eminence fracture fixation with high-strength suture, EndoButton, and suture anchor. Arthroscopy. (2012) 28:681–7. 10.1016/j.arthro.2011.10.026 22284410

[37] Li J Yu Y Liu C Su X Liao W Li Z. Arthroscopic fixation of tibial eminence fractures: a biomechanical comparative study of screw, suture, and suture anchor. Arthroscopy. (2018) 34:1608–16. 10.1016/j.arthro.2017.12.018 29397286

[38] Johnstone TM Baird DW Jr Cuellar-Montes A van Deursen WH Tompkins M Ganley TJ Screws or sutures? A pediatric cadaveric study of tibial spine fracture repairs. Am J Sports Med. (2023) 51:2589–95. 10.1177/03635465231181059 37382335

[39] Callanan M Allen J Flutie B Tepolt F Miller PE Kramer D Suture versus screw fixation of tibial spine fractures in children and adolescents: a comparative study. Orthop J Sports Med. (2019) 7:2325967119881961. 10.1177/2325967119881961 31803786 PMC6876177

[40] Jain S Modi P Dayma RL Mishra S. Clinical outcome of arthroscopic suture versus screw fixation in tibial avulsion of the anterior cruciate ligament in skeletally mature patients. J Orthop. (2023) 35:7–12. 10.1016/j.jor.2022.10.006 36325248 PMC9619313

[41] Osti L Buda M Soldati F Del Buono A Osti R Maffulli N. Arthroscopic treatment of tibial eminence fracture: a systematic review of different fixation methods. Br Med Bull. (2016) 118:73–90. 10.1093/bmb/ldw018 27151952 PMC5127426

[42] Chang CJ Huang TC Hoshino Y Wang CH Kuan FC Su WR Functional outcomes and subsequent surgical procedures after arthroscopic suture versus screw fixation for ACL tibial avulsion fractures: a systematic review and meta-analysis. Orthop J Sports Med. (2022) 10:23259671221085945. 10.1177/23259671221085945 35400137 PMC8990705

[43] Cruz AI Jr Lee RJ Kushare I Baghdadi S Green DW Ganley TJ Tibial spine fractures in young athletes. Clin Sports Med. (2022) 41:653–70. 10.1016/j.csm.2022.05.006 36210164

[44] Moran TE Werner BC. Surgery and rotator cuff disease: a review of the natural history, indications, and outcomes of nonoperative and operative treatment of rotator cuff tears. Clin Sports Med. (2023) 42:1–24. 10.1016/j.csm.2022.08.001 36375863

